# S100 Calcium-Binding Protein P and Cathepsin E as Key Mediators in Pancreatic Cancer Tumorigenesis

**DOI:** 10.3390/biomedicines13112780

**Published:** 2025-11-14

**Authors:** Yu Meng, Qian Deng, Ye Zhang, Fang Wei, Jun Wu, Haijiao Yan

**Affiliations:** Department of Oncology, The Third Affiliated Hospital of Soochow University, Changzhou 213000, China; mengyu20000206@126.com (Y.M.); dq939400375@163.com (Q.D.); zhangye13250612@163.com (Y.Z.); m15830810796@163.com (F.W.)

**Keywords:** pancreatic cancer, survival, invasion, epithelial–mesenchymal transition, PI3K–AKT signaling pathway

## Abstract

**Background/Objectives:** Pancreatic cancer (PC) remains one of the deadliest malignancies, with challenges that hinder early detection and few actionable molecular targets. In this study, we aimed to identify biomarkers predictive of PC to support its diagnosis and treatment. **Methods:** Proteins from formalin-fixed, paraffin-embedded pooled samples of PC (n = 15; 5 pools) and chronic pancreatitis (n = 10; 5 pools) tissues were analyzed via label-free quantitative proteomics using liquid chromatography-tandem mass spectrometry. Immunohistochemistry (IHC) was performed on PC tissue microarrays to assess S100 calcium-binding protein P (S100P) and cathepsin E (CTSE) expression (IHC evaluable pairs: n = 78 for S100P; n = 82 for CTSE). Transwell invasion assays were conducted to evaluate the effects of these proteins on PC cell invasiveness, and Western blotting was used to validate protein expression and elucidate associated molecular mechanisms. **Results:** Both S100P and CTSE were overexpressed in PC tissues compared with those in adjacent normal tissues. Elevated S100P expression correlated with poor prognosis, whereas higher CTSE expression predicted favorable outcomes; both served as independent prognostic factors in PC. Functionally, S100P promoted PC cell invasion, whereas CTSE suppressed it. Mechanistically, both proteins appeared to regulate epithelial–mesenchymal transition (EMT) and invasive capacity through activation or inhibition of the phosphoinositide 3-kinase (PI3K)–protein kinase B (AKT) signaling pathway. **Conclusions:** Elevated expression of S100P and CTSE in PC tissues serves as independent indicators in our model of patient survival. Both proteins regulate EMT and invasion, potentially via the PI3K–AKT pathway, and hold significant promise as prognostic biomarkers and therapeutic targets in PC.

## 1. Introduction

Pancreatic cancer (PC) ranks among the most lethal malignancies, exhibiting one of the highest mortality rates and a poor 5-year overall survival (OS) of only 13% [1,2]. Pancreatic ductal adenocarcinoma is the most prevalent subtype of PC. The poor prognosis is partly attributed to difficulties in diagnosing the disease at an early stage, which limits opportunities for curative surgical intervention [3,4]. Furthermore, the lack of effective molecular targets impedes therapeutic progress. Current treatment options, including surgery, chemotherapy, and radiotherapy, remain largely ineffective [5]. Therefore, identifying effective biomarkers and therapeutic targets for early-stage PC has become an urgent need.

The specific etiology of PC remains unclear. However, growing evidence indicates that patients with chronic pancreatitis (CP) have a significantly increased risk of developing PC [6]. Approximately 5 years after a CP diagnosis, the risk of PC increases by nearly eightfold [7]. The mechanisms by which CP promotes PC development are not fully understood, but it is hypothesized that a series of sequential events leads to progressive DNA damage, ultimately resulting in pancreatic intraepithelial neoplasia [8,9,10]. The persistent inflammatory and fibrotic microenvironment created by CP provides a crucial “soil” for PC development by activating key signaling pathways such as Kirsten rat sarcoma viral oncogene homolog and nuclear factor kappa-light-chain-enhancer of activated B cells (NF-κB) [11,12]. Numerous studies have shown that exacerbated pancreatic inflammation activates pancreatic stellate cells, potentially promoting carcinogenesis in genetically susceptible individuals [13,14]. Moreover, pathological studies have revealed a significantly higher incidence of high-grade pancreatic intraepithelial neoplasia (PanIN) in CP tissue adjacent to PC [15]. Several consensus guidelines have therefore classified patients with CP as a high-risk group for PC and recommend regular monitoring. Although these two conditions are clinically difficult to distinguish, recent research shows they are fundamentally distinct at the molecular level. For example, Wu et al. used machine learning to identify DNA methylation signatures in tissue and circulating cell-free DNA that accurately differentiate PC from CP [16]. In this complex transition from CP to PC, many molecular targets and signaling pathways remain unidentified, yet they may have significant implications for patient survival and prognosis.

S100P, a calcium-binding protein comprising 95 amino acids, belongs to the S100 family of proteins [17]. It is overexpressed in various malignancies, including colorectal [18,19], breast [20], lung [21], and liver cancers [22]. S100P plays a multifaceted role in tumor development, and its expression has been associated with poor outcomes in several gastrointestinal cancers. It contributes to tumor cell proliferation, survival, migration, invasion, and metastasis [18,19,22]. However, contradictory findings exist. A 2019 meta-analysis reported that S100P overexpression is associated with poor prognosis in hepatocellular carcinoma and cholangiocarcinoma but not consistently in pancreatic, gastric, colorectal, or gallbladder cancers [23]. Existing studies have shown that S100P can promote invasion and metastasis of PC through both intracellular and extracellular mechanisms. For instance, S100P secreted by PC cells can bind extracellularly to the receptor for advanced glycation end products (RAGE), activating the mitogen-activated protein kinase and NF-κB pathways to enhance invasion [24,25]. Intracellularly, S100P can activate Ezrin, thereby promoting the transendothelial migration of tumor cells [26]. S100P also plays a role in the epithelial–mesenchymal transition (EMT); studies have confirmed that it promotes EMT, migration, and invasion in colon cancer cells [27]. However, the relationship between S100P and EMT in PC remains unclear and warrants further investigation.

Cathepsin E (CTSE) is an intracellular aspartic protease that, along with pepsin A and cathepsin D, forms the peptidase A1 family [28]. Found in a limited range of cell types, CTSE is predominantly expressed in immune system cells such as lymphocytes, macrophages, microglia, and dendritic cells [29]. Its precise physiological functions remain unclear, as most studies have focused on its normal biological roles. For instance, Tsukuba et al. reported that CTSE-deficient mice spontaneously develop atopic dermatitis-like lesions, associated with increased bacterial infection and reduced conversion of interleukin (IL)-18 and IL-1β [30]. Moreover, CTSE-deficient mice on a high-fat diet exhibit impaired adipose tissue development due to reduced macrophage infiltration [31]. CTSE also plays an important role in antigen processing through the major histocompatibility complex (MHC) class II pathway [32]. Previous studies have shown that CTSE is selectively expressed in several malignant tumors, including lung, bladder, and esophageal cancers [33,34,35]. In PC, CTSE expression and proteolytic activity have been utilized to develop imaging probes for precise detection and activatable 5-ALA prodrugs [36]. Furthermore, suppression of CTSE expression has been linked to abnormal activation of the EMT pathway and poor prognosis in patients with breast cancer [37]. Although CTSE is believed to influence tumor invasion and EMT, its role and specific mechanisms in PC have not yet been elucidated.

Despite an incomplete understanding of the underlying cellular and molecular mechanisms, tumor invasion and metastasis remain major contributors to PC-related mortality. These processes are primarily driven by interactions between tumor cells and stromal components—a mechanism referred to as EMT [38]. During EMT, epithelial cells acquire a mesenchymal phenotype, resulting in enhanced motility and invasiveness that enable tumor cells to breach the basement membrane, invade surrounding tissues, and disseminate to distant sites. In individual cells, complete EMT occurs, while partial EMT is observed in cells migrating collectively [39]. Multiple signaling pathways regulate EMT, among which the phosphoinositide 3-kinase (PI3K)–protein kinase B (AKT) pathway is aberrantly activated during PC progression. This pathway is crucial in modulating malignant cell behavior and promoting EMT [40]. However, although the PI3K-AKT pathway’s involvement in EMT has been reported, it remains unclear whether S100P and CTSE regulate EMT via this pathway, underscoring the aim of this study.

In this study, we quantified differentially expressed proteins in formalin-fixed, paraffin-embedded (FFPE) tissues from PC and patients with CP, focusing on S100P and CTSE expression levels. We further validated their associations with clinicopathological features and patient prognosis, and assessed their effects on PC cell invasiveness to identify predictive biological markers for diagnosis and treatment.

## 2. Materials and Methods

### 2.1. Tissue Sample Preparation

Formalin-fixed, paraffin-embedded (FFPE) tissue sections from 15 pancreatic cancer (PC) and 10 patients with chronic pancreatitis (CP) were obtained from the Third Affiliated Hospital of Soochow University, Jiangsu, China. All cases were histologically confirmed as PC or CP. Initially, 15 PC and 10 CP FFPE samples were included. Due to limited tissue availability and concerns about protein yield and detection sensitivity when analyzed individually, sample pooling was employed. The 10 CP samples were randomly combined in groups of three to form five CP pools (A1–A5), and the 15 PC samples were randomly combined in groups of three to form five PC pools (B1–B5). Thus, the effective number of biological replicates was n = 5 for both CP and PC pools. For validation, patient tissue microarrays (TMAs) were purchased from Shanghai Outdo Biotech Company (Shanghai, China; product number: HPan-Ade180Sur-01), comprising 90 PC tissues and paired adjacent non-cancerous tissues. All procedures were conducted in accordance with the Declaration of Helsinki and were approved by the Ethics Committee of the Third Affiliated Hospital of Soochow University (Approval Number: [2024] Science No. 214).

### 2.2. Liquid Chromatography-Tandem Mass Spectrometry and Data Analysis

FFPE samples were deparaffinized in xylene and rehydrated through graded ethanol solutions. Proteins were extracted using SDT buffer (4% sodium dodecyl sulfate; Sangon Biotech, Shanghai, China) and 100 mM tris(hydroxymethyl)aminomethane hydrochloride (Tris-HCl) [pH 7.6]) and quantified using a bicinchoninic acid assay kit (Beyotime, Shanghai, China). For quality control, 20 μg of total protein from each sample was mixed with 6× sodium dodecyl sulfate–polyacrylamide gel electrophoresis (SDS-PAGE) loading buffer (Beyotime), boiled for 5 min, and resolved on 12% SDS-PAGE gels. Coomassie Brilliant Blue staining was used to evaluate electrophoretic band integrity. Only samples exhibiting clear, well-defined protein bands and sufficient total protein yield for at least two experimental replicates were used for further analysis. For mass spectrometry analysis, 80 μg of protein from quality-controlled samples was digested with trypsin using the filter-aided sample preparation method. The procedure included reduction with 100 mM dithiothreitol (Sigma-Aldrich, St. Louis, MO, USA) (boiling, 5 min), detergent removal using UA buffer (8 M urea [Bio-Rad, Hercules, CA, USA] in 0.1 M Tris-HCl, pH 8.5), alkylation with 100 mM iodoacetamide (Sigma-Aldrich) in UA buffer (30 min, room temperature, dark), and trypsin digestion using 4 μg sequencing-grade modified trypsin (Promega, Madison, WI, USA) at 37 °C for 16–18 h. Peptides were separated using an Easy nano-Liquid Chromatography 1200 Ultra-High-Performance Liquid Chromatography system (Thermo Fisher Scientific, Waltham, MA, USA) and analyzed on a Q Exactive Plus Hybrid Quadrupole-Orbitrap mass spectrometer (Thermo Fisher Scientific). Protein identification and label-free quantification were performed using MaxQuant (version 1.5.5.1). Statistical analyses were conducted in R (version 3.3.1) using the limma package. Proteins were considered significantly regulated when they met both criteria: adjusted *p* < 0.05 (FDR correction) and |log2 fold change| ≥ 1.5. Missing values were imputed using the “missing not at random” approach, randomly sampling from a left-shifted Gaussian distribution (1.8 standard deviation shift, 0.3 width).

### 2.3. Immunohistochemistry and Data Analysis

TMAs (HPan-Ade180Sur-01, Shanghai Outdo Biotech Co.) containing 90 paired PC and adjacent non-cancerous tissues were used for immunohistochemistry (IHC) analysis. TMAs were baked, deparaffinized, and subjected to antigen retrieval, followed by IHC staining according to standard procedures. Sections were incubated with primary antibodies: anti-*cathepsin E* (CTSE) (AF1294, R&D Systems, Minneapolis, MN, USA; dilution 1:50) and anti-S100P (AB133554, Abcam, Cambridge, UK; dilution 1:10,000). Corresponding secondary antibodies and diaminobenzidine (DAB) chromogen were used for visualization. Slides were independently evaluated by two blinded pathologists without access to clinical data. S100P and CTSE expression levels were quantified using H-scores (range: 0–300), calculated as:

H-score = intensity (0 = negative, 1 = weak, 2 = moderate, 3 = strong) × percentage of positive tumor cells (0–100%).

For discordant evaluations, a third blinded pathologist re-scored the slides, and final scores were determined by consensus. Of the 90 tissue pairs, 12 were excluded from S100P analysis and 8 from CTSE analysis due to suboptimal staining or insufficient tumor content, yielding 78 evaluable S100P and 82 evaluable CTSE pairs. Optimal cut-off values for survival analysis were determined via iterative Kaplan–Meier analysis to maximize discrimination in survival outcomes: S100P expression was classified as low (<180) or high (≥180), and CTSE expression as low (≤170) or high (>170).

### 2.4. Cell Sources and Culture

The PC cell lines PANC-1, BxPC-3, and AsPC-1 were obtained from the Cell Bank of the Chinese Academy of Sciences (Shanghai, China). The human pancreatic ductal epithelial cell line (HPDE) was kindly provided by the Second Affiliated Hospital of Sun Yat-sen University. BxPC-3 cells were cultured in RPMI 1640 medium (Hyclone, Logan, UT, USA) supplemented with 10% fetal bovine serum (FBS; Gibco, Thermo Fisher Scientific, Waltham, MA, USA) and 1% penicillin-streptomycin (Gibco). PANC-1 and HPDE cells were maintained in complete Dulbecco’s Modified Eagle Medium (DMEM; Hyclone) containing 10% FBS and 1% penicillin-streptomycin. All cell lines were routinely tested for mycoplasma contamination using a polymerase chain reaction (PCR)-based method targeting the 16S rRNA gene and were confirmed to be negative before use. The recommended passage ratios were as follows: PANC-1, 1:2–1:4; BxPC-3, 1:2–1:3 (or 1:3–1:6); and HPDE, 1:2–1:3. Cell line authentication for PANC-1 and BxPC-3 was verified using short tandem repeat (STR) profiling. All cell cultures were incubated at 37 °C in a humidified atmosphere containing 5% CO_2_.

### 2.5. Lentiviral Vector Construction and Transfection

Shanghai Junli Biotechnology Company (Shanghai, China) designed and constructed the S100P and CTSE overexpression plasmids, short hairpin RNA (shRNA) constructs targeting S100P and CTSE, and the corresponding negative control plasmids. The shRNA sequences used in the S100P and CTSE experiments are listed in Table 1. Transfection was performed following the manufacturer’s instructions using Lipofectamine 3000 (Thermo Fisher Scientific).

### 2.6. Quantitative Real-Time PCR

Total RNA was extracted from PC cells using TRIzol reagent (Thermo Fisher Scientific). Complementary DNA (cDNA) was generated through reverse transcription with the use of a PrimeScript RT kit from Beyotime. The cDNA was diluted and analyzed using quantitative real-time PCR (qRT-PCR) to assess messenger ribonucleic acid expression levels, with GAPDH used as the reference gene. The primer sequences used in the analysis are provided in Table 2.

### 2.7. Western Blot Analysis

Cells were lysed with lysis buffer (Beyotime) to extract total protein. Protein concentration was determined using the bicinchoninic acid assay (Beyotime), and equal amounts of protein were separated using SDS-PAGE. After electrophoresis, proteins were transferred onto membranes, blocked, and incubated sequentially with primary and secondary antibodies. Detection and visualization were performed using a Tanon-4600 automated chemiluminescence imaging system (Tanon, Shanghai, China). Quantitative analysis of protein band intensity was carried out using ImageJ software (version 1.53k). Details of the antibodies used are listed in Table 3.

### 2.8. Transwell Invasion Assay

The upper chamber of the Transwell insert was coated with diluted Matrigel (BD Biosciences, San Jose, CA, USA). A 100 µL cell suspension containing trypsinized and counted cells was added to the upper chamber, while 600 µL of culture medium supplemented with 20% FBS (Gibco) was placed in the lower chamber to serve as a chemoattractant. After 48 h of incubation at 37 °C in a humidified 5% CO_2_ atmosphere, non-migrated cells were gently removed with cotton swabs. The invaded cells were fixed with 4% paraformaldehyde (Beyotime), stained with 0.1% crystal violet (Beyotime), and counted under an inverted microscope (Leica Microsystems, Wetzlar, Germany).

### 2.9. Statistical Analysis

Differences in S100P and CTSE expression were analyzed using the Wilcoxon rank-sum test. The chi-squared test was used to assess associations between protein expression and clinicopathological characteristics. Kaplan–Meier survival curves were generated using the R packages “survival” and “survminer.” Univariate and multivariate Cox proportional hazards regression analyses were performed, with statistical significance set at *p* < 0.05. One-way analysis of variance was used to compare differences among groups. Statistical analyses were conducted using SPSS (version 26.0), and graphical outputs were generated using GraphPad Prism (version 9.5.1). Statistical significance was denoted as * *p* < 0.05, ** *p* < 0.01, and *** *p* < 0.001.

## 3. Results

### 3.1. Significant Difference Analysis of Protein Quantification Results in Formalin-Fixed, Paraffin-Embedded Tissues

**Figure 1:**
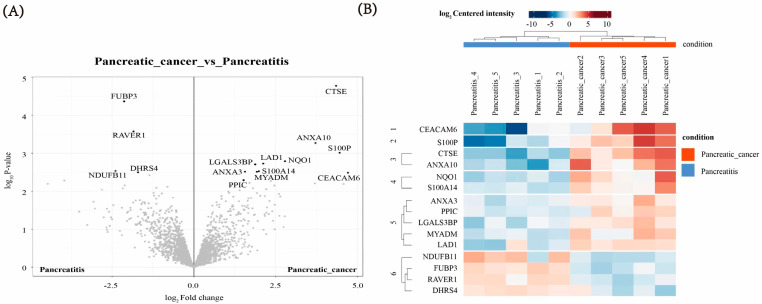
Differentially expressed proteins in pancreatic cancer (PC) and chronic pancreatitis (CP). (**A**) Volcano plot illustrating differential protein expression. Grey points represent non-significant proteins, whereas black points represent significantly differentially expressed proteins. (**B**) Heatmap showing the expression landscape of differential protein intensity in PC and CP. From left to right: five CP sample sets and five PC sample sets; from top to bottom: 11 proteins upregulated in PC and four proteins upregulated in CP.

MaxQuant analysis identified 2749 proteins that were reproducibly quantified. Data containing at least three non-missing values across five replicate experiments were selected for statistical analysis. Fifteen proteins were differentially expressed, with 11 upregulated and four downregulated in pancreatic cancer (PC) compared with those in chronic pancreatitis (CP). A volcano plot was generated to visualize differences in protein expression and associated *p*-values between groups, highlighting significant differential expression (Figure 1A). In addition, a heatmap revealed distinct expression patterns of the differentially expressed proteins across the tissue samples (Figure 1B). The proteins CEACAM6, S100P, cathepsin E (CTSE), ANXA10, NQO1, S100A14, ANXA3, PPIC, LGALS3BP, MYADM, LAD1, NDUFB11, FUBP3, RAVER1, and DHRS4 exhibited asymmetric distributions between PC and CP samples (Table 4).

### 3.2. S100P and CTSE Are Upregulated in Human PC

**Figure 2:**
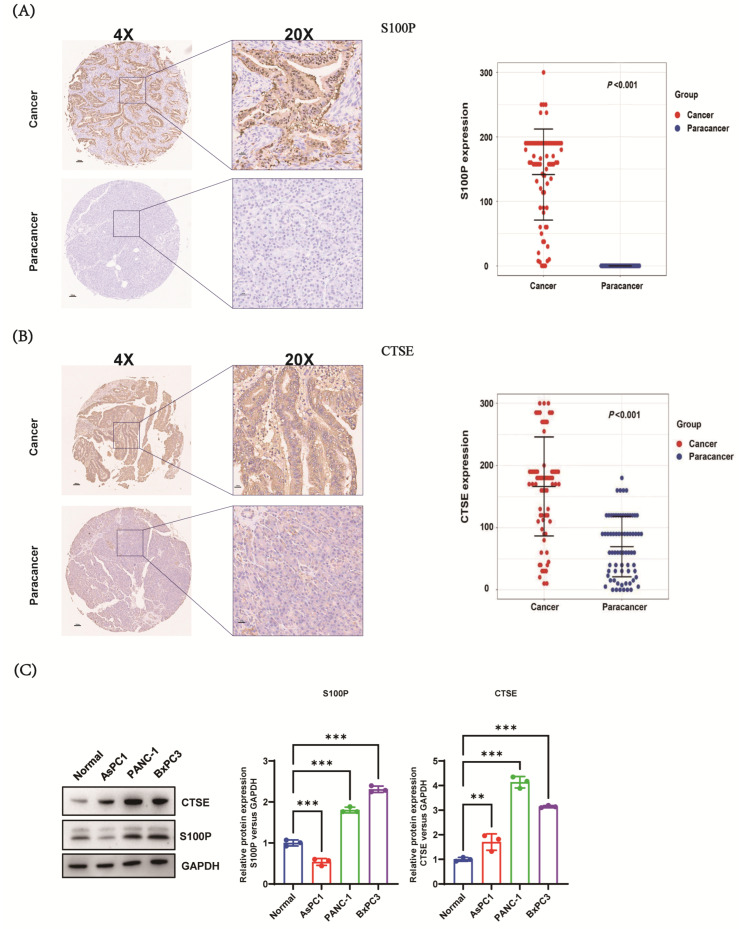
Overexpression of S100P and cathepsin E (CTSE) in human pancreatic cancer (PC) (n = 78 pairs for S100P; n = 82 pairs for CTSE in tissue microarrays; n = 3 for cell line experiments). (**A**,**B**) Expression of S100P (**A**) and CTSE (**B**) in tissue microarrays of PC. Representative images of PC tissue (**above**) and corresponding adjacent non-cancerous tissue (**below**) are shown (magnification, 20×). (**C**) Western blot analysis of S100P and CTSE expression in PC and normal pancreatic epithelial cell lines. ** *p* < 0.01, *** *p* < 0.001. S100P, S100 calcium-binding protein P.

Immunohistochemistry (IHC) analysis of the tissue microarray (TMA) showed that S100P was localized in the cytoplasm, cell membrane, and nucleus of malignant cells, with no detectable staining in adjacent non-cancerous tissues. In contrast, CTSE was primarily localized in the cytoplasm and, to a lesser extent, in the membrane and nucleus of malignant cells, showing weak expression in the cytoplasm and membrane of adjacent non-cancerous tissues. Of the 90 pairs of PC and adjacent non-cancerous tissues, 78 tumor and 78 adjacent tissue samples were analyzable after excluding sections with unsatisfactory staining. IHC scores for both S100P and CTSE were significantly higher in PC tissues than in adjacent non-cancerous tissues (*p* < 0.001; Figure 2A,B). Consistently, S100P and CTSE were overexpressed in PANC-1, BXPC-3, and AsPC-1 PC cell lines compared with those in the HPDE cell line (Figure 2C). The PANC-1 and BXPC-3 cell lines were subsequently selected for functional experiments.

### 3.3. Association Between S100P and CTSE Expression Levels and Clinicopathological Characteristics of Patients with PC

High S100P expression was significantly associated with tumor size (*p* = 0.001) but not with sex, age, T stage, N stage, M stage, or TNM stage (Table 5). In contrast, CTSE expression was significantly associated with tumor size (*p* < 0.001), T stage (*p* < 0.001), and M stage (*p* = 0.001), but not with sex, age, N stage, or overall TNM stage (Table 6).

### 3.4. Prognostic Significance of S100P and CTSE Expression Levels in Overall Survival (OS) Among Patients with PC

**Figure 3:**
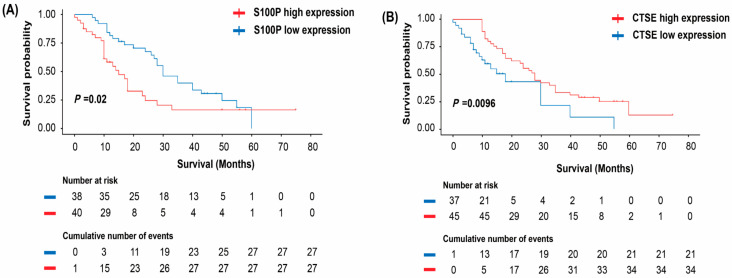
Association of S100P and cathepsin E (CTSE) expression with overall survival (OS) in patients with pancreatic cancer (PC). (**A**,**B**) Kaplan–Meier curves showing OS according to S100P (**A**) and CTSE (**B**) expression levels. The log-rank test was used to determine statistical significance. S100P, S100 calcium-binding protein P.

High S100P expression was significantly associated with poorer OS (*p* = 0.02; Figure 3A), whereas elevated CTSE expression correlated with improved OS (*p* = 0.0096; Figure 3B).

Univariate analysis for S100P revealed that tumor size (*p* = 0.001), N stage (*p* = 0.012), TNM stage (*p* = 0.031), and S100P expression (*p* = 0.024) were significantly associated with OS. Multivariate analysis of variables with *p* < 0.05 indicated that tumor size (*p* = 0.022) and S100P expression (*p* = 0.039) were independent prognostic factors for OS, whereas N stage and TNM stage were not significant (Table 7).

Similarly, univariate analysis for CTSE showed significant associations of tumor size (*p* = 0.001), N stage (*p* = 0.013), TNM stage (*p* = 0.029), and CTSE expression (*p* = 0.012) with OS. Multivariate analysis confirmed tumor size (*p* = 0.03) and CTSE expression (*p* = 0.019) as independent prognostic factors for OS, while N stage and TNM stage did not show significant associations (Table 8).

### 3.5. Effects of S100P and CTSE on the Invasion of PC Cells

**Figure 4:**
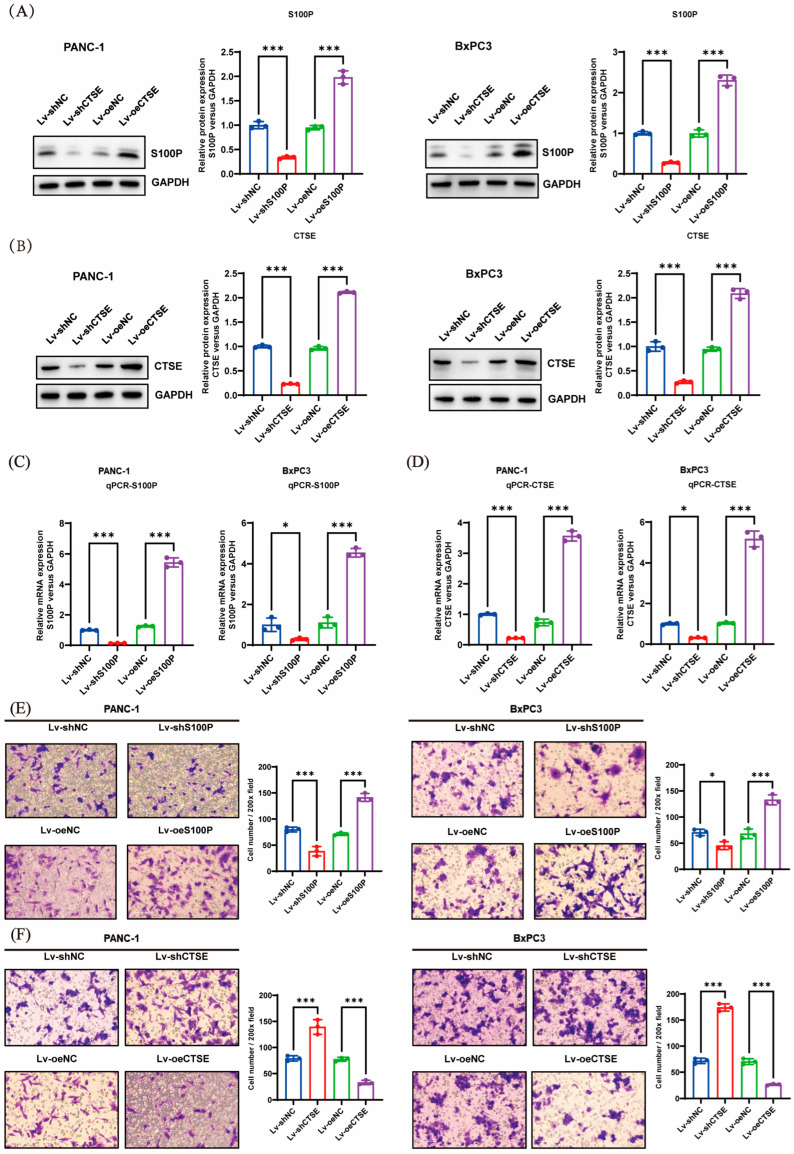
Effects of S100P and cathepsin E (CTSE) on pancreatic cancer (PC) cell invasion (n = 3 independent experiments). (**A**,**B**) Western blot analysis evaluating transfection efficiency of S100P (**A**) and CTSE (**B**) following knockdown or overexpression in PANC-1 and BxPC-3 cells. (**C**,**D**) quantitative real-time polymerase chain reaction (qRT-PCR) assessment of S100P (**C**) and CTSE (**D**) gene expression after knockdown or overexpression. (**E**,**F**) Transwell invasion assays showing changes in the invasive ability of PANC-1 and BxPC-3 cells after S100P (**E**) or CTSE (**F**) modulation. S100P, S100 calcium-binding protein P. * *p* < 0.05, *** *p* < 0.001.

The efficiency of S100P and CTSE knockdown and overexpression in PANC-1 and BxPC-3 cell lines was confirmed using Western blotting (Figure 4A,B) and quantitative real-time polymerase chain reaction (qRT-PCR) (Figure 4C,D). Overexpression of S100P markedly enhanced PC cell invasiveness, whereas S100P knockdown reduced invasive capacity (Figure 4E). Conversely, CTSE overexpression significantly inhibited PC cell invasion, while CTSE knockdown substantially increased invasion in vitro (Figure 4F).

### 3.6. S100P and CTSE Regulate Phosphoinositide 3-Kinase (PI3K)–Protein Kinase B (AKT) Signaling Pathways and Affect Epithelial–Mesenchymal Transition (EMT)

**Figure 5:**
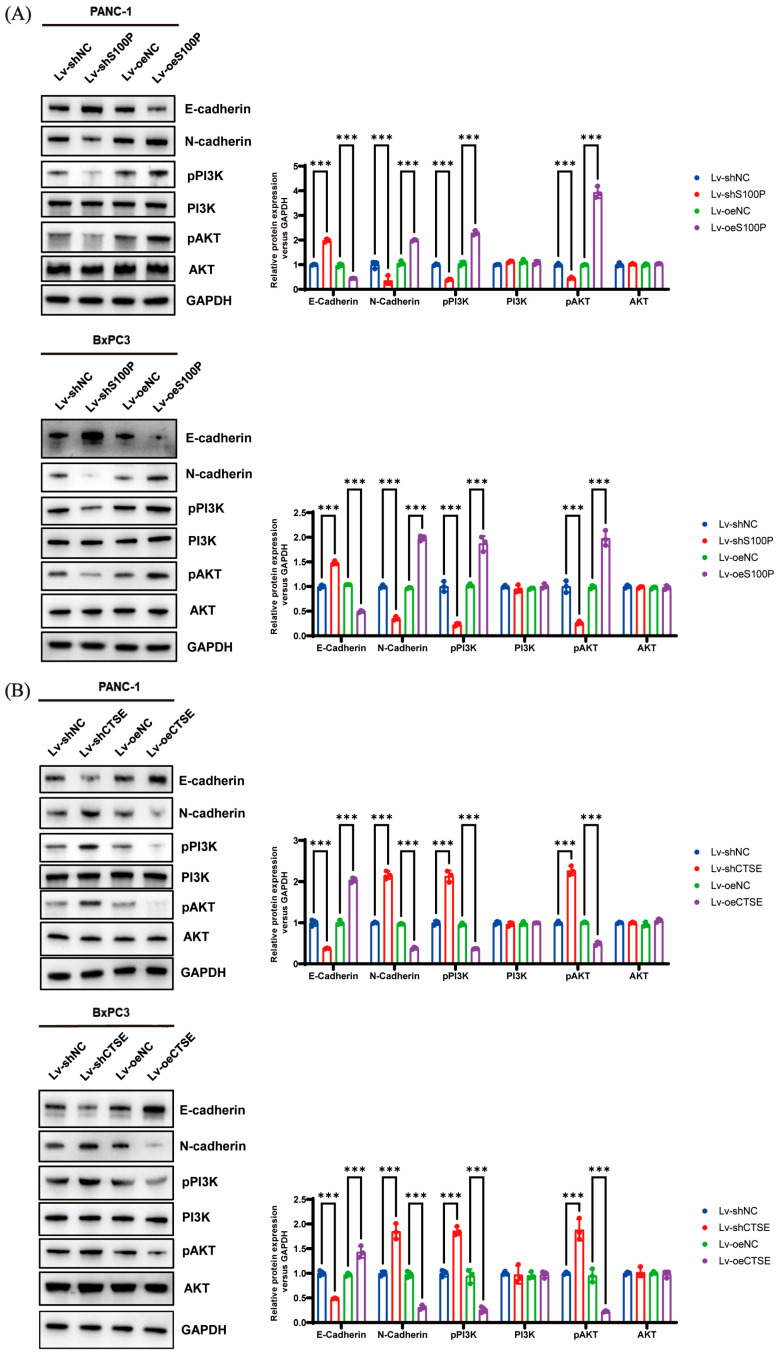
S100P and cathepsin E (CTSE) regulate the phosphoinositide 3-kinase (PI3K)–protein kinase B (AKT) signaling pathways and affect Epithelial–Mesenchymal Transition (EMT) in pancreatic cancer (PC) cells (n = 3 independent experiments). (**A**,**B**) Western blot analysis of E-cadherin, N-cadherin, p-PI3K, total PI3K, p-AKT, and total AKT in PANC-1 and BxPC-3 cells following S100P (**A**) or CTSE (**B**) knockdown or overexpression. *** *p* < 0.001. S100P, S100 calcium-binding protein P.

Following S100P knockdown, levels of N-cadherin, p-PI3K, and p-AKT were significantly reduced, while E-cadherin expression increased. Conversely, S100P overexpression produced the opposite pattern (Figure 5A). Similarly, CTSE downregulation resulted in elevated p-AKT, p-PI3K, and N-cadherin levels, along with reduced E-cadherin expression, whereas CTSE overexpression reversed these effects (Figure 5B). Total levels of PI3K and AKT were not significantly altered by S100P and CTSE expression changes (Figure 5A,B).

## 4. Discussion

Pancreatic cancer (PC) is an exceedingly aggressive malignancy with a high tendency for early metastasis. Despite ongoing advancements in therapeutic strategies, survival outcomes for patients with PC remain unsatisfactory [1,41]. Consequently, elucidating the molecular mechanisms underlying tumor progression and identifying novel biomarkers for diagnosis, therapy, and prognosis are crucial for improving overall survival (OS) in patients with PC.

In this study, we conducted a comprehensive proteomic analysis of PC and chronic pancreatitis (CP) tissues. Our results revealed that S100P and cathepsin E (CTSE) were significantly overexpressed in PC tissues relative to CP tissues, suggesting their potential roles in the pathogenesis and progression of PC. To validate the expression and clinical significance of S100P and CTSE in PC, we analyzed tissue microarrays (TMAs) containing samples from 90 patients with PC and paired adjacent non-cancerous tissues, performing immunohistochemistry (IHC) to assess the expression levels of these proteins.

S100P was localized within the cytoplasm, cell membrane, and nucleus of PC cells, whereas it was undetectable in adjacent non-cancerous tissues. Further analysis revealed that S100P expression in PC tissues was significantly higher than in adjacent tissues. These findings align with previous research indicating that S100P is not only markedly overexpressed in PC but also closely associated with the progression of pancreatic intraepithelial neoplasia (PanIN) grades [42]. IHC analysis of CTSE revealed predominant cytoplasmic expression in PC cells, with lower levels in the cell membrane and nucleus, while adjacent non-cancerous tissues exhibited weak cytoplasmic and membranous staining. Data analysis confirmed that CTSE expression was elevated in PC tissues compared with that in adjacent non-cancerous tissues. Our findings corroborate those of Cruz-Monserrate et al., who reported that CTSE is significantly overexpressed in PC and serves as an early biological marker for PanIN [43]. Collectively, these results suggest that the progression from CP to PanIN and subsequently to PC may involve multiple stages, during which the concentrations of S100P and CTSE accumulate, potentially playing important biological roles in PC development. Correlation analysis demonstrated a significant association between S100P expression and tumor size in patients with PC. CTSE expression was also correlated with tumor size, as well as T and M stages.

Building on these findings, we evaluated the prognostic significance of S100P and CTSE expression in patients with PC. Elevated S100P expression was significantly associated with poorer OS, while tumor size, N stage, TNM stage, and S100P expression emerged as key prognostic factors. Multivariate analysis identified tumor size and S100P expression as independent prognostic indicators. Conversely, patients with positive CTSE expression showed significantly better survival than those with negative expression. Univariate analysis further demonstrated significant associations among N stage, TNM stage, CTSE expression, and prognosis, while multivariate analysis confirmed tumor size and CTSE expression as independent prognostic factors. Collectively, these results indicate that S100P and CTSE are strongly associated with the prognosis of PC. Nevertheless, the precise mechanisms through which they contribute to PC pathogenesis and progression remain to be clarified.

During tumor progression, epithelial–mesenchymal transition (EMT) serves as a critical early event that promotes the invasion and dissemination of cancer cells. This process involves molecular and phenotypic changes, notably reduced epithelial marker expression and increased mesenchymal marker levels [44]. EMT is characterized by several hallmark features, including the loss of cell polarity, morphological alterations, cytoskeleton remodeling, reduced cell adhesion, and the acquisition of invasive and migratory capabilities [45]. In various malignancies, including PC, the phosphoinositide 3-kinase (PI3K)–protein kinase B (AKT) signaling pathway is activated by multiple components such as cytokines, chemokines, and growth factors. Activation of this pathway plays a key role in regulating essential cellular processes, including growth, survival, proliferation, metabolism, and motility [46]. Numerous studies have established a strong association between PI3K-AKT pathway activation and EMT. AKT can modulate the cell cycle during EMT by downregulating E-cadherin [47]. Moreover, AKT functions as a crucial downstream effector of methylsterol monooxygenase 1, influencing EMT through its interactions with epidermal growth factor receptor and various cross-talk proteins within the PI3K/AKT/mTOR signaling cascade [48].

We knocked down or overexpressed S100P and CTSE to examine their effects on the invasive capacity of PC cells. Our experimental results showed that S100P downregulation inhibited invasion, whereas its overexpression enhanced invasive ability. In contrast, CTSE knockdown promoted PC cell invasion, while overexpression suppressed it. This contrasting behavior may be attributed to distinct underlying signaling mechanisms. Further mechanistic investigations demonstrated that S100P overexpression promoted EMT and invasion via activation of the PI3K–AKT pathway, whereas elevated CTSE expression inhibited both EMT and invasion by suppressing this same pathway. Although these results appear contradictory, we propose that they highlight the multifaceted role of CTSE within the complex tumor microenvironment of PC. Increasing evidence suggests that protein expression levels in tumor tissues represent a composite signal, and their prognostic implications are highly dependent on cellular origin. Our IHC and survival data reflect the relationship between total CTSE levels within tumor tissue and patient prognosis. We hypothesize that CTSE is primarily expressed and secreted by infiltrating immune cells (such as tumor-associated macrophages and lymphocytes), meaning that high CTSE expression reflects a more anti-tumor immune state that effectively suppresses tumor progression and metastasis, leading to improved OS. Thus, while CTSE expression within cancer cells may have inhibitory effects on invasion, its overall high tissue level correlates with a favorable prognosis. This tumor microenvironment-defined prognostic biomarker pattern is gaining increasing recognition [49]. High CTSE expression (likely derived mainly from immune cells) signifies an active anti-tumor immune response and is associated with better prognosis. Moreover, CTSE acts as an intrinsic regulator within cancer cells, exerting cell-autonomous effects that inhibit invasion and metastasis. Taken together, we propose that CTSE plays a context-dependent role in the pathogenesis of PC: in the early stages, CTSE within cancer cells may limit invasion and migration by suppressing the PI3K-AKT pathway and EMT, and in later stages, CTSE expression by immune cells in the tumor microenvironment may increase total CTSE levels, thereby serving as a favorable prognostic indicator.

We speculate that CTSE and S100P establish a delicate “balance” within the tumor microenvironment. This dynamic equilibrium enables PC cells to survive and adapt under varying microenvironmental and physiological conditions by flexibly modulating the relative expression of CTSE and S100P, thereby influencing tumor progression and metastasis. Although both S100P and CTSE are highly expressed in pancreatic tissue, they predict different prognostic outcomes, suggesting distinct responses to cancer treatment that may ultimately affect patient survival. This phenomenon is also commonly observed in clinical practice. In recent years, immunotherapy—particularly immune checkpoint inhibitors—has achieved major breakthroughs in oncology. However, its efficacy in PC remains unsatisfactory, primarily due to the unique immunosuppressive microenvironment of this malignancy [50]. Notably, the EMT process is closely linked to the formation of an immunosuppressive microenvironment: tumor cells undergoing EMT often exhibit characteristics of “immune-cold tumors,” including poor infiltration of effector T cells and accumulation of immunosuppressive cells such as myeloid-derived suppressor cells (MDSCs) [51]. The heterogeneity of the tumor microenvironment and mechanisms of immune escape are central reasons for the marked variability in immunotherapy responses [52], making the selection of appropriate “treatment timing” a critical issue in precision oncology. A global informatics study showed that since 2020, research focus has shifted notably toward neoadjuvant immunotherapy [53]. Drawing from the experience in chronic myeloid leukemia, where early-stage (chronic phase) treatment with imatinib yields near-curative outcomes, whereas late-stage (blast crisis) therapy has limited efficacy, early intervention in solid tumors such as PC—before extensive clonal evolution and establishment of an immunosuppressive microenvironment—may be key to improving therapeutic success [54]. Based on this, we propose a significant scientific hypothesis: the balance between S100P and CTSE may influence the immune microenvironment of PC by regulating the EMT process. Specifically, S100P-driven EMT may foster an immunosuppressive milieu by recruiting MDSCs and promoting M2 macrophage polarization, leading to resistance to immunotherapy. Conversely, CTSE may counteract this process, maintaining a more favorable immune environment, which could explain its association with a good prognosis.

Although neither our study nor available public databases (such as The Cancer Genome Atlas [TCGA]) revealed a significant linear correlation between the messenger ribonucleic acid expression levels of S100P and CTSE—implying no direct transcriptional regulation—this does not preclude the possibility of more complex, indirect interactions at the protein-functional level. For instance, S100P has been shown to act as a secreted protein that binds to the cell surface receptor RAGE, thereby activating downstream pathways such as PI3K/AKT. CTSE, an aspartic protease, likely has intracellular substrates and signaling regulatory networks that remain incompletely understood. These two proteins may operate as a finely tuned “balance,” maintaining dynamic equilibrium in PC progression by independently modulating shared downstream pathways, such as PI3K/AKT, or interacting nodes within this axis, ultimately determining the tumor’s invasive phenotype. S100P, as a secreted oncoprotein, represents a potential target for therapeutic intervention (e.g., monoclonal antibodies). Blocking its function may effectively inhibit the PI3K/AKT pathway and reverse EMT. Conversely, high CTSE expression is associated with a favorable prognosis and exhibits inhibitory effects on both the PI3K/AKT pathway and cellular invasion, making it a reliable prognostic biomarker. In the future, the expression ratio of CTSE to S100P could be incorporated into prognostic assessment models to enhance the accuracy of patient outcome prediction.

Currently, carbohydrate antigen 19-9 (CA19-9) is the only serum biomarker approved by the U.S. FDA for managing PC. However, its specificity is limited, as it can also be elevated in benign conditions such as cholangitis and pancreatitis; hence, it is not recommended for early screening in asymptomatic individuals [55]. Our study demonstrated that both S100P and CTSE are significantly overexpressed in PC tissues, with IHC analysis confirming their diagnostic value in distinguishing malignant from adjacent non-cancerous tissues. In terms of prognosis, high S100P expression correlated with poorer OS, whereas high CTSE expression correlated with improved survival. Multivariate analysis confirmed both as independent prognostic factors. Notably, a recent study on intrahepatic cholangiocarcinoma revealed co-localization of CTSE^+^ tumor cells with specific tumor-associated macrophages, jointly contributing to poor patient prognosis. This finding suggests that CTSE serves not only as a diagnostic marker but also as a prognostic indicator intricately linked to the tumor immune microenvironment [56]. Such features make CTSE functionally complementary to CA19-9, which is primarily used for monitoring treatment response.

Modern oncology is shifting from reliance on single biomarkers toward integrated, multi-parameter diagnostic models. A promising approach is to combine novel biomarkers with established indicators like CA19-9 to construct predictive algorithms. Yang et al. developed a liquid biopsy technique that simultaneously detected CA19-9 and Kirsten rat sarcoma viral oncogene homolog mutations, achieving a diagnostic accuracy of 92% for pancreatic ductal adenocarcinoma—substantially outperforming single-marker assays [57]. This provides a clear translational direction for our work: combining S100P and/or CTSE with CA19-9 could yield a highly sensitive and specific diagnostic panel. Furthermore, comprehensive scoring systems developed using logistic regression or machine learning algorithms may further enhance diagnostic performance.

This study has some limitations. First, we employed PC TMAs to examine the expression of S100P and CTSE and their associations with clinicopathological parameters. However, the sample size was relatively small, and some IHC slices were excluded due to poor quality or absence of tumor tissue, thereby reducing the number of analyzable samples. To strengthen and validate our findings, it would be valuable to assess additional public cohorts (e.g., TCGA-PAAD) or employ a second TMA for independent validation. Future studies should therefore include larger, multicenter cohorts to obtain more robust conclusions. Second, in our cellular experiments, we primarily investigated the effects of S100P and CTSE on invasion and EMT in relation to the PI3K–AKT signaling pathway. Given the complexity of tumor biology, future studies should explore additional regulatory mechanisms. Specifically, future research should involve PI3K inhibitors (such as LY294002 or Wortmannin) and AKT inhibitors (such as MK-2206) in cell models with S100P/CTSE overexpression or knockdown to confirm the upstream role of this signaling axis. Another limitation of this study lies in the incomplete assessment of the EMT process. While we examined the expression changes of E-cadherin and N-cadherin, we did not cover other key EMT markers such as Vimentin, Snail, Slug, and ZEB1, nor did we analyze the subcellular localization of β-catenin. Future research should systematically evaluate these markers and, in conjunction with techniques such as immunofluorescence, clarify their cellular distribution to more comprehensively reveal the roles of S100P and CTSE in EMT regulation. In the functional experiments of this study, although multiple independent shRNAs were used and consistent phenotypes were observed, future rescue experiments will further confirm the specificity of S100P and CTSE function. This will be one of the key directions of our subsequent research. Future research can further utilize wound healing experiments and 3D spheroid culture models to investigate the effects of S100P and CTSE on cell migration and multicellular invasive behaviors across different dimensions, which will help comprehensively reveal their complex roles in pancreatic cancer metastasis. Finally, due to limited tissue availability and concerns about low yield, insufficient detection sensitivity, and potential interference from tissue heterogeneity when performing protein extraction from small formalin-fixed, paraffin-embedded samples, we used a pooling strategy—combining tissues from multiple patients for proteomic analysis. While this approach improved protein detection, it also introduced potential confounding of inter-patient biological variability. Therefore, the proteomic differences identified between PC and chronic pancreatitis in this study should be regarded as preliminary and hypothesis-generating rather than confirmatory. The core value of this study lies in providing a valuable list of candidate proteins for further investigation. Future studies should rigorously validate these candidate biomarkers in larger, independent cohorts, using patient-specific analyses and more refined sampling methods such as laser microdissection.

## 5. Conclusions

The expression levels of S100P and cathepsin E (CTSE) were significantly higher in pancreatic cancer (PC) tissues than in adjacent normal tissues. Elevated S100P expression was associated with poor prognosis in patients with PC, whereas increased CTSE expression correlated with favorable outcomes. Both proteins served as independent prognostic factors in our survival model. Functionally, S100P enhanced the invasive capacity of PC cells, while CTSE inhibited invasion. Their regulatory effects on epithelial–mesenchymal transition (EMT) and cell invasion may be mediated through the phosphoinositide 3-kinase (PI3K)–protein kinase B (AKT) signaling pathway. Overall, S100P and CTSE show considerable potential as prognostic biomarkers and therapeutic targets in PC.

## Figures and Tables

**Table 1:** Lentivirus sequences used for S100P and cathepsin E (CTSE).

Name	Sequence
shS100P-1	GGACTTCAGTGAGTTCATATTCAAGAGATATGAACTCACTGAAGTCCTTTTTT
shS100P-2	CCTGTCACAAGTACTTTGATTCAAGAGATCAAAGTACTTGTGACAGGTTTTTT
shS100P-3	CCGTGGATAAATTGCTCAATTCAAGAGATTGAGCAATTTATCCACGGTTTTTT
shCTSE-1	GCCCTTCCGACAAGATTAATTCAAGAGATTAATCTTGTCGGAAGGGCTTTTTT
shCTSE-2	CCAGCAGTTTGGAGAAAGTTTCAAGAGAACTTTCTCCAAACTGCTGGTTTTTT
shCTSE-3	GTGCCAACCTTAACGTCATTTCAAGAGAATGACGTTAAGGTTGGCACTTTTTT

S100P, S100 calcium-binding protein P.

**Table 2:** Primer design for quantitative real-time polymerase chain reaction (qRT-PCR).

Gene	Forward (5′-3′)	Reverse (5′-3′)
CTSE	ACATCCAGGTGGGAGGCACT	AGGTTGGCACACTCCACAGC
S100P	CAGACCCTGACCAAGGGGGA	CTGGGCATCTCCATTGGCGT
GAPDH	GCGGGGCTCTCCAGAACATC	TCCACCACTGACACGTTGGC

CTSE, cathepsin E; S100P, S100 calcium-binding protein P.

**Table 3:** Antibodies used in this study.

Antibody	Manufacturer	Catalog Number
CTSE	Abclonal	A22092
S100P	Abclonal	A20795
E-Cadherin	Proteintech	20874-1-AP
N-Cadherin	CST	13116S
pPI3K	CST	17366S
PI3K	CST	4249S
pAKT	CST	4060S
AKT	CST	2920S
Anti-rabbit IgG, HRP-linked Antibody	CST	7074
GAPDH (D16H11) Rabbit mAb (HRP Conjugate)	CST	8884

CTSE, cathepsin E; PI3K, phosphoinositide 3-kinase; AKT, protein kinase B; Ig, immunoglobulin; S100P, S100 calcium-binding protein P.

**Table 4:** Differentially expressed proteins between pancreatic cancer (PC) and chronic pancreatitis (CP).

Gene Name	PC vs. CP log2 Fold Change	Adjusted *p*-Value
CEACAM6	4.71	0.0215
S100P	4.46	0.0023
CTSE	4.35	<0.0001
ANXA10	3.72	0.0004
NQO1	2.79	0.0073
LAD1	2.12	0.0091
S100A14	1.99	0.0193
MYADM	1.92	0.0202
LGALS3BP	1.88	0.0097
ANXA3	1.56	0.0200
PPIC	1.52	0.0487
DHRS4	−1.71	0.0209
RAVER1	−1.84	<0.0001
FUBP3	−2.11	<0.0001
NDUFB11	−2.40	0.0189

CTSE, cathepsin E; S100P, S100 calcium-binding protein P.

**Table 5:** Correlation between S100P expression and clinicopathological characteristics in patients with pancreatic cancer (PC) (n = 78).

Clinical Pathological Characteristics	n	S100P Expression Level		χ^2^	*p*
		Low	High		
Sex				2.956	0.086
Male	50	28	22		
Female	28	10	18		
Age (years)				0.195	0.658
<60	37	19	18		
≥60	41	19	22		
Tumor size (cm)				11.496	**0.001**
<4	36	25	11		
≥4	42	13	29		
T stage				0.795	0.373
T1–T2	55	25	30		
T3–T4	23	13	10		
N stage				0.082	0.744
N0	48	24	24		
N1–N3	30	14	16		
M stage				0.031	0.860
M0	63	31	32		
M1	15	7	8		
TNM stage				0.244	0.621
I	33	15	18		
II–IV	45	23	22		

Boldfacing indicates significant *p* values. S100P, S100 calcium-binding protein P.

**Table 6:** Correlation between cathepsin E (CTSE) expression and clinicopathological characteristics in patients with pancreatic cancer (PC) (n = 82).

Clinical Pathological Characteristics	n	CTSE Expression Level		χ^2^	*p*
		Low	High		
Sex				0.046	0.831
Male	52	23	29		
Female	30	14	16		
Age (years)				0.071	0.791
<60	39	17	22		
≥60	43	20	23		
Tumor size (cm)				16.569	**<0.001**
<4	38	8	30		
≥4	44	29	15		
T stage				23.981	**<0.001**
T1–T2	56	15	41		
T3–T4	26	22	4		
N stage				0.204	0.651
N0	51	24	27		
N1–N3	31	13	18		
M stage				10.478	**0.001**
M0	66	24	42		
M1	16	13	3		
TNM stage				0.012	0.913
I	36	16	20		
II–IV	46	21	25		

Boldfacing indicates significant *p* values.

**Table 7:** Univariate and multivariate analyses of S100P expression and overall survival (OS) in patients with pancreatic cancer (PC) (n = 78).

Factors	Univariate Analysis			Multivariate Analysis		
	HR	95% CI	*p* Value	HR	95% CI	*p* Value
Sex (male/female)	1.397	0.778–2.509	0.262			
Age (≥60/<60 years)	0.944	0.553–1.614	0.835			
Tumor size (≥4/<4 cm)	2.539	1.434–4.496	**0.001**	2.049	1.108–3.79	**0.022**
T (T3–T4/T1–T2)	1.35	0.748–2.439	0.319			
N (N1–N3/N0)	2.005	1.164–3.452	**0.012**	1.545	0.752–3.176	0.237
M (M1/M0)	1.409	0.74–2.682	0.297			
TNM (II–IV/I)	1.881	1.06–3.336	**0.031**	1.247	0.572–2.719	0.579
S100P (high/low)	1.881	1.088–3.252	**0.024**	1.825	1.032–3.226	**0.039**

Boldfacing indicates significant *p* values. HR, hazard ratio; CI, confidence interval; S100P, S100 calcium-binding protein P.

**Table 8:** Univariate and multivariate analyses of cathepsin E (CTSE) expression and overall survival (OS) in patients with pancreatic cancer (PC) (n = 82).

Factors	Univariate Analysis			Multivariate Analysis		
	HR	95% CI	*p* Value	HR	95% CI	*p* Value
Sex (male/female)	1.392	0.777–2.494	0.266			
Age (≥60/<60 years)	0.938	0.552–1.594	0.814			
Tumor size (≥4/<4 cm)	2.567	1.457–4.523	**0.001**	1.964	1.067–3.616	**0.03**
T (T3–T4/T1–T2)	1.295	0.718–2.337	0.39			
N (N1–N3/N0)	1.982	1.155–3.403	**0.0** **13**	1.717	0.834–3.536	0.142
M (M1/M0)	1.358	0.714–2.583	0.35			
TNM (II–IV/I)	1.87	1.064–3.286	**0.029**	1.215	0.566–2.612	0.617
CTSE (high/low)	0.482	0.271–0.854	**0.012**	0.476	0.256–0.884	**0.019**

Boldfacing indicates significant *p* values. HR, hazard ratio; CI, confidence interval.

## Data Availability

The original contributions presented in this study are included in the article. Further inquiries can be directed to the corresponding author.

## Abbreviations

The following abbreviations are used in this manuscript:

PC	Pancreatic cancer
IHC	Immunohistochemistry
S100P	S100 calcium-binding protein P
CTSE	Cathepsin E
EMT	Epithelial–mesenchymal transition
OS	Overall survival
CP	Chronic pancreatitis
PanIN	Pancreatic intraepithelial neoplasia
PI3K	Phosphoinositide 3-kinase
AKT	Protein kinase B
TMA	Tissue microarray
TGCA	The Cancer Genome Atlas
IL	Interleukin
FFPE	Formalin-fixed, paraffin-embedded
SDS-PAGE	Sodium dodecyl sulfate–polyacrylamide gel electrophoresis
Tris-HCL	Tris(hydroxymethyl)aminomethane hydrochloride

## References

[1] Stoop T.F., Javed A.A., Oba A., Koerkamp B.G., Seufferlein T., Wilmink J.W., Besselink M.G. (2025). Pancreatic cancer. Lancet.

[2] Kotecha K., Tree K., Ziaziaris W.A., McKay S.C., Wand H., Samra J., Mittal A. (2024). Centralization of Pancreaticoduodenectomy: A Systematic Review and Spline Regression Analysis to Recommend Minimum Volume for a Specialist Pancreas Service. Ann. Surg..

[3] Latenstein A.E.J., van der Geest L.G.M., Bonsing B.A., Groot Koerkamp B., Haj Mohammad N., de Hingh I., de Meijer V.E., Molenaar I.Q., van Santvoort H.C., van Tienhoven G. (2020). Nationwide trends in incidence, treatment and survival of pancreatic ductal adenocarcinoma. Eur. J. Cancer.

[4] Connor A.A., Gallinger S. (2022). Pancreatic cancer evolution and heterogeneity: Integrating omics and clinical data. Nat. Rev. Cancer.

[5] Wood L.D., Canto M.I., Jaffee E.M., Simeone D.M. (2022). Pancreatic Cancer: Pathogenesis, Screening, Diagnosis, and Treatment. Gastroenterology.

[6] Wu H., Gao S.Z., Yin L.D., Wang Y.H., Deng S.J., Lu Z.P., Jin G., Zou W.B. (2025). Germline mutations and pancreatic cancer risk in chronic pancreatitis. Gut.

[7] Kirkegard J., Mortensen F.V., Cronin-Fenton D. (2017). Chronic Pancreatitis and Pancreatic Cancer Risk: A Systematic Review and Meta-analysis. Am. J. Gastroenterol..

[8] Das A., Bararia A., Mukherjee S., Sikdar N. (2024). Chronic pancreatitis as a driving factor for pancreatic cancer: An epidemiological understanding. World J. Clin. Oncol..

[9] Le Cosquer G., Maulat C., Bournet B., Cordelier P., Buscail E., Buscail L. (2023). Pancreatic Cancer in Chronic Pancreatitis: Pathogenesis and Diagnostic Approach. Cancers.

[10] Chen X., Liu F., Xue Q., Weng X., Xu F. (2021). Metastatic pancreatic cancer: Mechanisms and detection. Oncol. Rep..

[11] Shi C., Pan F.C., Kim J.N., Washington M.K., Padmanabhan C., Meyer C.T., Kopp J.L., Sander M., Gannon M., Beauchamp R.D. (2019). Differential Cell Susceptibilities to *Kras^G12D^* in the Setting of Obstructive Chronic Pancreatitis. Cell Mol. Gastroenterol. Hepatol..

[12] Liou G.Y., Döppler H., Necela B., Krishna M., Crawford H.C., Raimondo M., Storz P. (2013). Macrophage-secreted cytokines drive pancreatic acinar-to-ductal metaplasia through NF-κB and MMPs. J. Cell Biol..

[13] Tao Y., Shao F., Cai M., Liu Z., Peng Y., Huang Q., Meng F. (2021). Activated Pancreatic Stellate Cells Enhance the Warburg Effect to Cause the Malignant Development in Chronic Pancreatitis. Front. Oncol..

[14] Wang Z., Dong S., Zhou W. (2024). Pancreatic stellate cells: Key players in pancreatic health and diseases. Mol. Med. Rep..

[15] Hwang I.K., Kim H., Lee Y.S., Kim J., Cho J.Y., Yoon Y.S., Han H.S., Hwang J.H. (2015). Presence of pancreatic intraepithelial neoplasia-3 in a background of chronic pancreatitis in pancreatic cancer patients. Cancer Sci..

[16] Wu Y., Seufert I., Al-Shaheri F.N., Kurilov R., Bauer A.S., Manoochehri M., Moskalev E.A., Brors B., Tjaden C., Giese N.A. (2023). DNA-methylation signature accurately differentiates pancreatic cancer from chronic pancreatitis in tissue and plasma. Gut.

[17] Crnogorac-Jurcevic T., Missiaglia E., Blaveri E., Gangeswaran R., Jones M., Terris B., Costello E., Neoptolemos J.P., Lemoine N.R. (2003). Molecular alterations in pancreatic carcinoma: Expression profiling shows that dysregulated expression of S100 genes is highly prevalent. J. Pathol..

[18] Schmid F., Dahlmann M., Röhrich H., Kobelt D., Hoffmann J., Burock S., Walther W., Stein U. (2022). Calcium-binding protein S100P is a new target gene of MACC1, drives colorectal cancer metastasis and serves as a prognostic biomarker. Br. J. Cancer.

[19] Lin M., Fang Y., Li Z., Li Y., Feng X., Zhan Y., Xie Y., Liu Y., Liu Z., Li G. (2021). S100P contributes to promoter demethylation and transcriptional activation of SLC2A5 to promote metastasis in colorectal cancer. Br. J. Cancer.

[20] Cong Y., Cui Y., Wang S., Jiang L., Cao J., Zhu S., Birkin E., Lane J., Ruge F., Jiang W.G. (2020). Calcium-Binding Protein S100P Promotes Tumor Progression but Enhances Chemosensitivity in Breast Cancer. Front. Oncol..

[21] Gao L., Bai Y., Zhou J., Liang C., Dong Y., Han T., Liu Y., Guo J., Wu J., Hu D. (2024). S100P facilitates LUAD progression via PKA/c-Jun-mediated tumor-associated macrophage recruitment and polarization. Cell. Signal..

[22] Qi L.N., Ma L., Wu F.X., Chen Y.Y., Xing W.T., Jiang Z.J., Zhong J.H., Chen Z.S., Gong W.F., Ye J.Z. (2021). S100P as a novel biomarker of microvascular invasion and portal vein tumor thrombus in hepatocellular carcinoma. Hepatol. Int..

[23] Liu B.X., Tang C.T., Dai X.J., Zeng L., Cheng F., Chen Y., Zeng C. (2021). Prognostic Value of S100P Expression in Patients With Digestive System Cancers: A Meta-Analysis. Front. Oncol..

[24] Arumugam T., Simeone D.M., Van Golen K., Logsdon C.D. (2005). S100P promotes pancreatic cancer growth, survival, and invasion. Clin. Cancer Res..

[25] Ji Y.F., Huang H., Jiang F., Ni R.Z., Xiao M.B. (2014). S100 family signaling network and related proteins in pancreatic cancer (Review). Int. J. Mol. Med..

[26] Koltzscher M., Neumann C., König S., Gerke V. (2003). Ca2+-dependent binding and activation of dormant ezrin by dimeric S100P. Mol. Biol. Cell.

[27] Zuo Z., Zhang P., Lin F., Shang W., Bi R., Lu F., Wu J., Jiang L. (2018). Interplay between Trx-1 and S100P promotes colorectal cancer cell epithelial-mesenchymal transition by up-regulating S100A4 through AKT activation. J. Cell. Mol. Med..

[28] Pontious C., Kaul S., Hong M., Hart P.A., Krishna S.G., Lara L.F., Conwell D.L., Cruz-Monserrate Z. (2019). Cathepsin E expression and activity: Role in the detection and treatment of pancreatic cancer. Pancreatology.

[29] Zaidi N., Hermann C., Herrmann T., Kalbacher H. (2008). Emerging functional roles of cathepsin E. Biochem. Biophys. Res. Commun..

[30] Tsukuba T., Okamoto K., Okamoto Y., Yanagawa M., Kohmura K., Yasuda Y., Uchi H., Nakahara T., Furue M., Nakayama K. (2003). Association of cathepsin E deficiency with development of atopic dermatitis. J. Biochem..

[31] Kadowaki T., Kido M.A., Hatakeyama J., Okamoto K., Tsukuba T., Yamamoto K. (2014). Defective adipose tissue development associated with hepatomegaly in cathepsin E-deficient mice fed a high-fat diet. Biochem. Biophys. Res. Commun..

[32] Bennett K., Levine T., Ellis J.S., Peanasky R.J., Samloff I.M., Kay J., Chain B.M. (1992). Antigen processing for presentation by class II major histocompatibility complex requires cleavage by cathepsin E. Eur. J. Immunol..

[33] Yang Y., Wang M., Liu B. (2018). Exploring and comparing of the gene expression and methylation differences between lung adenocarcinoma and squamous cell carcinoma. J. Cell. Physiol..

[34] Chen H., Yang W., Li Y., Ma L., Ji Z. (2023). Leveraging a disulfidptosis-based signature to improve the survival and drug sensitivity of bladder cancer patients. Front. Immunol..

[35] Fisher O.M., Levert-Mignon A.J., Lord S.J., Botelho N.K., Freeman A.K., Thomas M.L., Falkenback D., Wettstein A., Whiteman D.C., Bobryshev Y.V. (2015). High Expression of Cathepsin E in Tissues but Not Blood of Patients with Barrett’s Esophagus and Adenocarcinoma. Ann. Surg. Oncol..

[36] Abd-Elgaliel W.R., Cruz-Monserrate Z., Wang H., Logsdon C.D., Tung C.H. (2013). Pancreatic cancer-associated Cathepsin E as a drug activator. J. Control. Release.

[37] Kawakubo T., Yasukochi A., Toyama T., Takahashi S., Okamoto K., Tsukuba T., Nakamura S., Ozaki Y., Nishigaki K., Yamashita H. (2014). Repression of cathepsin E expression increases the risk of mammary carcinogenesis and links to poor prognosis in breast cancer. Carcinogenesis.

[38] Allgayer H., Mahapatra S., Mishra B., Swain B., Saha S., Khanra S., Kumari K., Panda V.K., Malhotra D., Patil N.S. (2025). Epithelial-to-mesenchymal transition (EMT) and cancer metastasis: The status quo of methods and experimental models 2025. Mol. Cancer.

[39] Majidpoor J., Mortezaee K. (2021). Steps in metastasis: An updated review. Med. Oncol..

[40] Guo Z., Ashrafizadeh M., Zhang W., Zou R., Sethi G., Zhang X. (2023). Molecular profile of metastasis, cell plasticity and EMT in pancreatic cancer: A pre-clinical connection to aggressiveness and drug resistance. Cancer Metastasis Rev..

[41] Martin R.F. (2024). Management of Pancreatic Cancer. Surg. Clin. N. Am..

[42] Sato N., Fukushima N., Matsubayashi H., Goggins M. (2004). Identification of maspin and S100P as novel hypomethylation targets in pancreatic cancer using global gene expression profiling. Oncogene.

[43] Cruz-Monserrate Z., Abd-Elgaliel W.R., Grote T., Deng D., Ji B., Arumugam T., Wang H., Tung C.H., Logsdon C.D. (2012). Detection of pancreatic cancer tumours and precursor lesions by cathepsin E activity in mouse models. Gut.

[44] Cho E.S., Kang H.E., Kim N.H., Yook J.I. (2019). Therapeutic implications of cancer epithelial-mesenchymal transition (EMT). Arch. Pharm. Res..

[45] Bracken C.P., Goodall G.J. (2022). The many regulators of epithelial-mesenchymal transition. Nat. Rev. Mol. Cell Biol..

[46] Li O., Li L., Sheng Y., Ke K., Wu J., Mou Y., Liu M., Jin W. (2023). Biological characteristics of pancreatic ductal adenocarcinoma: Initiation to malignancy, intracellular to extracellular. Cancer Lett..

[47] Tang H., Massi D., Hemmings B.A., Mandalà M., Hu Z., Wicki A., Xue G. (2016). AKT-ions with a TWIST between EMT and MET. Oncotarget.

[48] Cao R., Zhang Z., Tian C., Sheng W., Dong Q., Dong M. (2022). Down-regulation of MSMO1 promotes the development and progression of pancreatic cancer. J. Cancer.

[49] Binnewies M., Roberts E.W., Kersten K., Chan V., Fearon D.F., Merad M., Coussens L.M., Gabrilovich D.I., Ostrand-Rosenberg S., Hedrick C.C. (2018). Understanding the tumor immune microenvironment (TIME) for effective therapy. Nat. Med..

[50] Bailey P., Chang D.K., Forget M.A., Lucas F.A., Alvarez H.A., Haymaker C., Chattopadhyay C., Kim S.H., Ekmekcioglu S., Grimm E.A. (2016). Exploiting the neoantigen landscape for immunotherapy of pancreatic ductal adenocarcinoma. Sci. Rep..

[51] Bagati A., Kumar S., Jiang P., Pyrdol J., Zou A.E., Godicelj A., Mathewson N.D., Cartwright A.N.R., Cejas P., Brown M. (2021). Integrin αvβ6-TGFβ-SOX4 Pathway Drives Immune Evasion in Triple-Negative Breast Cancer. Cancer Cell.

[52] Zhang M., Liu C., Tu J., Tang M., Ashrafizadeh M., Nabavi N., Sethi G., Zhao P., Liu S. (2025). Advances in cancer immunotherapy: Historical perspectives, current developments, and future directions. Mol. Cancer.

[53] Guo S.B., Hu L.S., Huang W.J., Zhou Z.Z., Luo H.Y., Tian X.P. (2024). Comparative investigation of neoadjuvant immunotherapy versus adjuvant immunotherapy in perioperative patients with cancer: A global-scale, cross-sectional, and large-sample informatics study. Int. J. Surg..

[54] Wahida A., Buschhorn L., Fröhling S., Jost P.J., Schneeweiss A., Lichter P., Kurzrock R. (2023). The coming decade in precision oncology: Six riddles. Nat. Rev. Cancer.

[55] Tempero M.A., Malafa M.P., Al-Hawary M., Behrman S.W., Benson A.B., Cardin D.B., Chiorean E.G., Chung V., Czito B., Del Chiaro M. (2021). Pancreatic Adenocarcinoma, Version 2.2021, NCCN Clinical Practice Guidelines in Oncology. J. Natl. Compr. Cancer Netw..

[56] Fan G., Tao C., Li L., Xie T., Tang L., Han X., Shi Y. (2025). The co-location of MARCO+ tumor-associated macrophages and CTSE+ tumor cells determined the poor prognosis in intrahepatic cholangiocarcinoma. Hepatology.

[57] Yang Z., LaRiviere M.J., Ko J., Till J.E., Christensen T., Yee S.S., Black T.A., Tien K., Lin A., Shen H. (2020). A Multianalyte Panel Consisting of Extracellular Vesicle miRNAs and mRNAs, cfDNA, and CA19-9 Shows Utility for Diagnosis and Staging of Pancreatic Ductal Adenocarcinoma. Clin. Cancer Res..

